# Performance and variability of QuantiFERON Gold Plus assay associated with phlebotomy type

**DOI:** 10.1371/journal.pone.0207892

**Published:** 2018-11-19

**Authors:** Saroochi Agarwal, Duc T. Nguyen, Justin D. Lew, Edward A. Graviss

**Affiliations:** Department of Pathology and Genomic Medicine, Houston Methodist Research Institute, Houston, Texas, United States of America; University of Southampton Faculty of Medicine, UNITED KINGDOM

## Abstract

**Background:**

QuantiFERON Gold Plus (Plus) assay has two approved methods for blood collection: direct in-tube (Plus direct) or the transfer of blood from a lithium heparin tube (Plus transfer). Currently, there is little data comparing the results of Plus and the QuantiFERON Gold In-Tube (Gold) based on blood collection.

**Methods:**

In 2017, high risk healthcare workers undergoing annual tuberculosis infection screening at Houston Methodist Hospital, a private hospital in the Texas Medical Center (Houston, TX, U.S.A.) were consented and enrolled in a study comparing the Gold-in-tube (Gold), Plus direct in-tube, and Plus transfer assays. Blood was drawn concurrently for all 3 assays.

**Results:**

Phlebotomy occurred on 300 consecutive, consented and enrolled participants in the study. The proportion of positive test results for the Gold, Plus direct and Plus transfer assays were 10% (29/300), 12% (35/299) and 17% (51/299), respectively. The agreement in the results of Gold versus Plus direct, Gold versus Plus transfer, and Plus direct versus Plus transfer was 91%, kappa (κ) = 0.56; 91%, κ = 0.59; and 85%, κ = 0.37, respectively.

**Conclusions:**

Among high risk healthcare workers in a low prevalence tuberculosis setting, the Gold Plus assay had a higher proportion of positive results than the Gold in-tube assay. The agreement between the Gold, Plus direct and Plus transfer assays was unexpectedly low for simultaneously obtained samples. Blood transfer using lithium heparin offers individual clinics and public health programs greater ability to customize protocols, but variability of results still exists.

## Introduction

There are several diagnostic and screening assays for tuberculosis (TB) infection (TBI) including the tuberculin skin test (TST) and interferon gamma release assays (IGRAs), which include the QuantiFERON-TB Gold in-tube (QFT-G) (QIAGEN, Germantown, MD, USA) and T-SPOT.*TB* (Oxford Immunotec, Inc., Marlborough, MA, USA) assays. A new IGRA, QuantiFERON-TB Gold Plus (QFT-P) (QIAGEN, Germantown, MD, USA), was approved by the U.S.A. Food and Drug Administration (FDA) approved for use in the United States (U.S.) on June 8, 2017. Major differences between the QFT-G and the QFT-P include the removal of TB7.7 peptides, the addition of a second antigen tube containing shorter peptides for ESAT-6 and CFP-10, aimed at eliciting a response from CD8+ T-cells, as wells as the peptides directed at CD4+ cells in the first antigen tube, and the standardization of both blood collection and laboratory procedures (as described below).

The QFT-P is approved for a direct in-tube phlebotomy draw or an indirect phlebotomy draw into a lithium heparin (LiHp) tube, where the blood is subsequently transferred into the four QFT-P tubes. This standardized transfer procedure is expected to reduce indeterminate results caused by pre-analytical errors such as tubes not being shaken as the transfer will be conducted in the laboratory by trained technicians [1]. The second standardization is to bring uniformity to all labs by using a four point standard curve rather than an eight point standard curve, which is needed to calibrate and interpret optical density values into IFN-γ concentrations.

QFT-P is expected to be more sensitive than QFT-G; however, early publications on the sensitivity of QFT-P has shown equal sensitivity compared to QFT-G. Studies conducted in Japan, Italy, Germany, Belgium, and the Netherlands (low TB prevalence countries) found no significant differences between the sensitivity among bacteriologically and non- bacteriologically confirmed active TB patients and specificity among healthy subjects with low or no risk for TB between the third generation (QFT-G) and fourth generation (QFT-P) assays [2–7]. A study conducted among U.S. Health Care Workers (HCWs) found a positivity rate of 4% in the study population when using QFT-G and 6% when using QFT-P with 96% agreement between the assays [8].

Sensitivity and specificity are difficult to calculate in TBI assays because of the unavailability of a “Gold Standard” for latent TBI (LTBI) [9]. Surrogate measures are often used including active TB disease which may underestimate sensitivity of a test to detect TBI and overestimate specificity when using individuals with zero TB exposure risk. Specificity is usually estimated in IGRA /TST negative low risk individuals with no known exposures to TB diseased patients; however, when assessing TBI diagnostic performance, results from active TB patients and individuals with no TB risk factors are considered lower in the hierarchy of standards than correlation of results to the exposure gradient of TB infection [10, 11].

The goals of the current study were to: (1) Analyze the agreement and performance between the QFT-G and the QFT-P in a population of U.S. HCWs with greater than average risk, and (2) compare agreement and performance of QFT-P results based on different phlebotomy methods: directly collecting blood into the assay tubes (QFT-PD), and transferring the blood from a single standard heparinized tube into the QFT-P assay tubes (QFT-PT).

## Methods

Eligible HCWs at the Houston Methodist Hospital (HMH), a private hospital located in the Texas Medical Center in Houston, TX, U.S.A., undergoing annual TB screening were consented for participation in the study. Participants completed a short questionnaire on TB risk factors (including questions on demographic factors, employment and medical history) and had blood drawn by well-trained phlebotomists at the HMH Outpatient Laboratory. The study was approved by the HMH IRB (Pro00016966).

### Eligibility

HCWs were eligible for the study if they (1) had a previous positive tuberculin skin test (TST), (2) were foreign born, (3) had received a BCG vaccination, or (4) had immunosuppression due to a medical condition or medication. Exclusion criteria included: (1) not being eligible for a QFT-G test during annual TB screening (i.e. managers tested on a different cycle), (2) having a history of active TB disease or (3) having had a TST within three months of enrollment into the study (new employees).

### Blood collection

Participants had a total of 10mL of blood collected: 1.0mL directly drawn into each of the three QFT-G tubes (Grey, Red, and Purple), 1.0mL directly drawn into each of the two QFT-P antigen tubes (QFT-PD—Yellow and Green) and 5.0mL drawn into a LiHp blood. A single positive and negative control was used for both the QFT-G and QFT-PD assays. The blood tubes were transferred to the TB laboratory within 10 hours of collection and incubated at 37°C, meeting the manufacturers guidelines of initiating incubation within 16 hours of blood collection [12]. In the laboratory, the heparinized blood was transferred within 3 hours into of the 4 QFT-P tubes, which were subsequently incubated within two hours of the transfer [13].

### Sample processing and storage

Per the manufacturer’s protocols, all QFT tubes (QFT-G, QFT-PD, QFT-PT) were incubated between 16 and 24 hours at 37°C before being stored at room temperature until centrifugation [12]. QFT-G assays were run within 3 days of blood collection. QFT-P plasma was harvested and stored at -80°C before being batched and tested. An eight point standard curve was used to calibrate and interpret optical density into estimated IFN-γ for the QFT-G, but a four point standard curve was used for calibration and interpretation for the QFT-P assays. Excess unused plasma from the positive and negative control of the QFT-G assay were frozen and stored until the QFT-PD was run. The plasma was thawed along with the stored and frozen QFT-P plasma, and the plasma from the positive control, negative control, TB1 and TB2 tubes were run with the QFT-PD assay.

### Statistical analysis

Sample size was calculated to detect a 2% increase in the proportion of positive tests in the QFT-P assay compared to the QFT-G assay using a two-sided test if the proportion of positive QFT-G assays was 6.5%. As this was a pilot study, 10% of the sample size was enrolled in the study [14]. Frequencies and proportions of test results (positive, negative, and indeterminate) were calculated for the QFT-G and the two QFT-P assays. The agreements between QFT-G and QFT-PD, QFT-G and QFT-PT, and QFT-PD and QFT-PT were analyzed using percent agreement, Cohen’s kappa of inter-rater agreement (κ). The conservative cutoff of 0.7 IU/mL IFN-γ was utilized due to reproducibility studies identifying measurements between 0.2 and 0.7 IU/mL as being a “zone of uncertainty” where one is most likely to see reversions and conversions in serial testing [15]. Frequencies and proportions of QFT-P assay’s positive due to TB1, TB2 or both TB1 and TB2 with IFN-γ values greater than or above 0.35 IU/ml after subtracting the IFN-γ measured in the negative were reported as this is the manufacturer’s cutoff. Boxplots were used to compare absolute difference between TB1-nil or TB2 –nil against the cutoff of 0.35. The frequency and proportions of QFT-P assay’s positive due to TB1, TB2 or both TB1 and TB2 with IFN-γ values greater than 0.70 IU/mL were reported. Risk factors for having a positive assay result were identified using univariate and multiple logistic regression. Risk factors were defined as having a prior positive TST or IGRA, previous treatment for TBI, history of autoimmune disease(s), taking immunosuppressive drugs, or receiving a vaccine within 6 weeks prior to having the QFT. All analyses were conducted using SAS 9.4 (Cary, NC). A *P* < 0.05 was considered statistically significant.

## Results

### Characteristics of study participants

**Fig 1:**
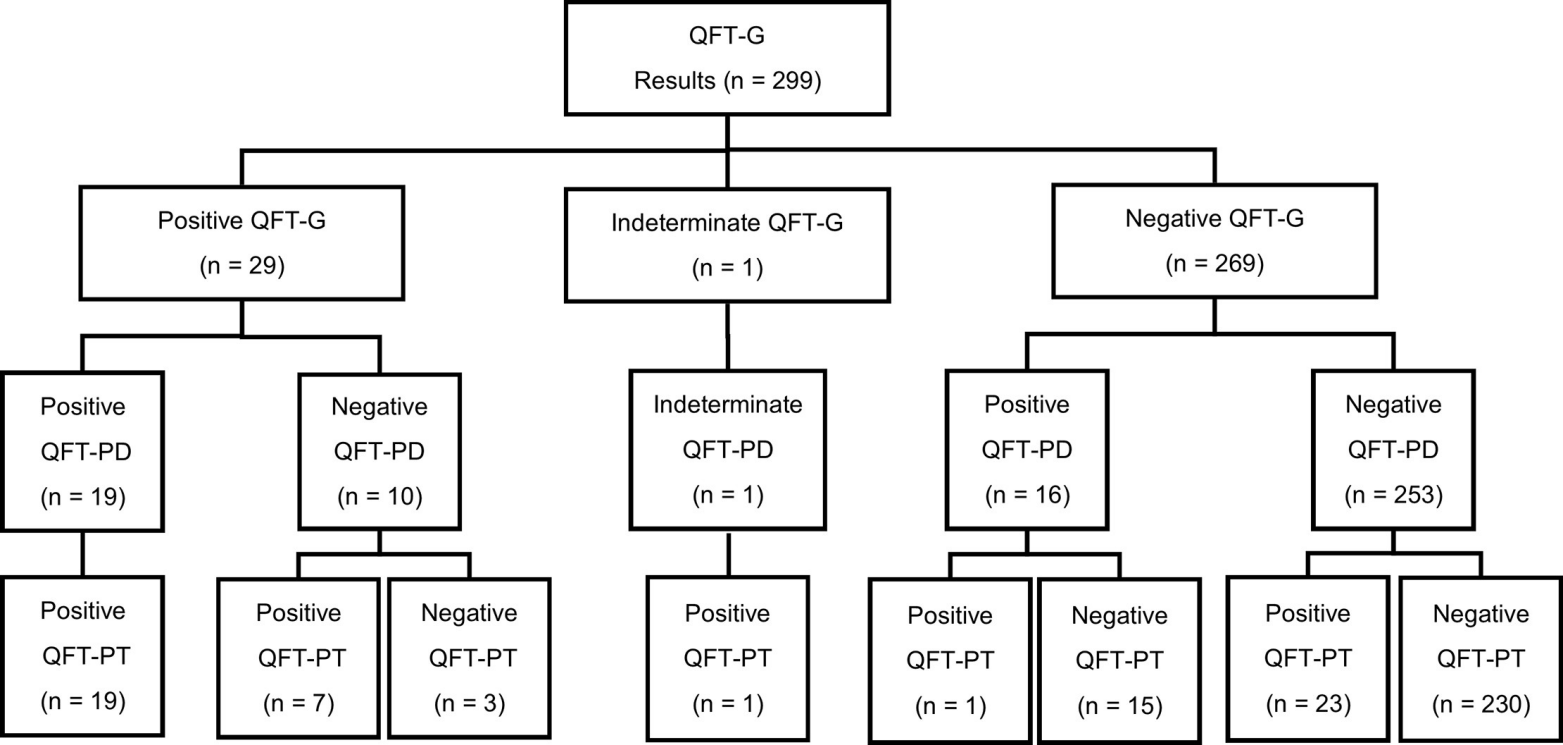
Flowchart of results from QFT-G, QFT-PD, and QFT-PT assay.

Of 300 enrolled participants, 280 (93%) completed the questionnaire and 299 had results for the two QFT-P assays. One participant had too little blood drawn to complete the QFT-P assay (Fig 1). Participants’ median age was 38 years (IQR: 31, 50). Participants were predominantly female (68%), Asian (45%), non-Hispanic (84%), foreign-born (79%), BCG vaccinated (66%), and had a previously positive TST (55%). Twenty-eight (10%) of 280 study participants who completed the questionnaires reported having received treatment for TBI prior to enrollment. Another eight percent (23/280) of participants reported having diabetes and two participants reported having HIV infection (Table 1).

**Table 1:** Demographic and clinical characteristics of participants with blood samples and completed questionnaires (N = 280).

Covariate	n	%
Age, median (IQR)	38	(31, 50)
Age		
> 30	62	22.3%
31–64	208	74.8%
65+	8	2.9%
Missing	2	
Gender		
Female	190	68.1%
Male	89	31.9%
Missing	1	
Pregnant		
Yes	7	3.2%
No	174	95.1%
Don't know/Prefer not to answer	2	1.1%
Missing	7	
Ethnicity		
White	74	27.1%
AA/Black	48	17.6%
Asian	123	45.1%
Other	25	9.2%
Don't know/Prefer not to answer	3	1.1%
Missing	7	
Hispanic		
Yes	42	15.1%
No	232	83.5%
Don't know/Prefer not to answer	4	1.4%
Missing	2	
US-Born		
Yes	55	20.7%
No	211	79.3%
Missing	14	
Received BCG Vaccine		
Yes	181	65.6%
No	69	25.0%
Don't know/Prefer not to answer	26	9.4%
Missing	4	
Result Last TST		
Positive	132	55.2%
Negative	92	38.5%
Don't know/Prefer not to answer	15	6.3%
Missing	41	
Result Last TB Blood Test		
Positive	10	4.4%
Negative	190	84.4%
Don't know/Prefer not to answer	25	11.1%
Missing	55	
Ever taken Treatment for TBI		
Yes	28	10.2%
No	241	87.6%
Don't know/Prefer not to answer	6	2.2%
Missing	5	
Medical Conditions		
Diabetes	23	8.3%
Autoimmune	14	5.1%
HIV	2	0.7%
Organ Transplant	1	0.4%
Kidney Dialysis	1	0.4%
Cancer	1	0.4%
Medications		
Corticosteroids	8	2.9%
TNF, alpha blockers, infliximab, etanercept, certolizumab	3	1.1%
Other Immunosuppressive therapy	4	1.5%
Smoke		
Yes	7	2.5%
No	270	97.5%
Missing	3	
Vaccine within 6 weeks		
Yes	37	13.5%
No	234	85.4%
Don't know/Prefer not to answer	3	1.1%
Missing	6	
Viral illness within 6 weeks		
Yes	11	4.0%
No	265	95.3%
Don't know/Prefer not to answer	2	0.7%
Missing	2	
Regularly consume green tea		
Yes	73	26.2%
No	203	72.8%
Don't know/Prefer not to answer	3	1.1%
Missing	1	

### Assay results and agreement

Twenty-nine of 299 (10%) participants had positive results on the QFT-G assay and one (0.3%) participant had an indeterminate result (Fig 1). The QFT-PD had 35 (12%) positive results and one indeterminate result, and the QFT-PT had 51 (17%) positive results and no indeterminate results (Fig 1). The percent agreement between the qualitative results of QFT-G and QFT-PD was 91% (κ = 0.56; Table 2). Of the 26 discordant results between QFT-G and QFT-PD, 16 (62%) had negative QFT-G and positive QFT-PD results. The percent agreement between QFT-G and the QFT-PT was 91% (κ = 0.59; Table 2). Of the 28 discordant results between the QFT-G and the QFT-PT, 24 (86%) had negative QFT-G test results and positive QFT-PD test results. The percent agreement was lowest between the QFT-PD and the QFT-PT (85%; κ = 0.37, Table 2). There was a significant difference in the proportion of results between the QFT-PD and QFT-PT (*P*<0.001).

**Table 2:** Agreement of QFT-G, QFT-PD, and QFT-PT and Comparison of QFT-P assay IFN-γ values by blood collection method.

QFT Results				
Overall (N = 299)	% agreement	95% CI	kappa	95% CI
**Cutoff Positive ≥0.35 IU/mL**				
QFT Gold versus QFT+ Direct	91	88–94	0.56	0.41–0.71
QFT Gold versus QFT+ Transfer	91	87–94	0.59	0.46–0.73
QFT+ Direct versus QFT+ Transfer	85		80–89	0.37
**Cutoff Positive >0.7 IU/mL**				** **
QFT Gold versus QFT+ Direct	93	90–96	0.55	0.37–0.72
QFT Gold versus QFT+ Transfer	94	90–96	0.55	0.37–0.73
QFT+ Direct versus QFT+ Transfer	92	87–95	0.5	0.32–0.67
**TST Positive (N = 131)**				
QFT Gold versus QFT+ Direct	93	87–97	0.72	0.55–0.89
QFT Gold versus QFT+ Transfer	90	84–95	0.61	0.43–0.80
QFT+ Direct versus QFT+ Transfer	86	79–92	0.51	0.32–0.71

Of the participants that reported the results of their last TST and had results for all three QFT assays (*n* = 238), 131 (55%) had a positive test result on their last TST. Eight of the 10 participants with a previous positive QFT-G reported having a previous positive TST, and the remaining two participants with previous positive QFT-G assays did not know the results of their last TST. In TST (+) participants, the agreements [%; κ] between pairs of assays were: QFT versus QFT-PD [93%; κ = 0.55], QFT-G versus QFT-PT [90%; κ = 0.55], and QFT-PD versus QFT-PT [86%; κ = 0.50] (Table 2).

**Fig 2:**
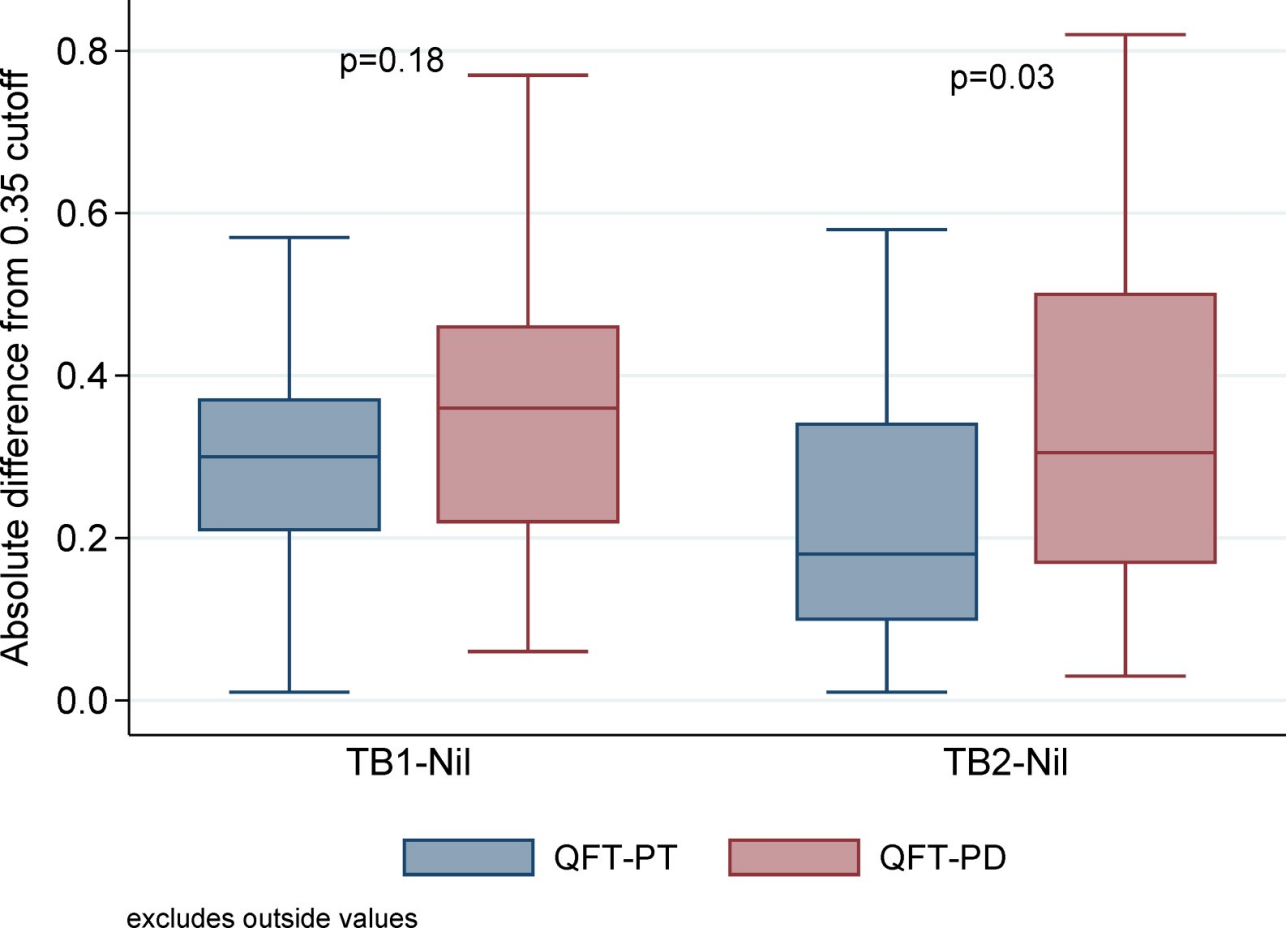
Absolute difference of TB1/TB2 minus Nil from the cut-off of 0.35, median (IQR) for discordant QFT-P results. *One patient had an indeterminate QFT-PD result that was converted to a negative result for this analysis.

Of the 46 discordant results between QFT-PD and QFT-PT, 30 (65%) had negative QFT-PD and positive QFT-PT results, and 15 (33%) QFT-PD with positive results were negative with QFT-PT (Fig 1). Nine (30%) of the 30 negative QFT-PD and positive QFT-PT results would remain positive with QFT-PT if the cutoff was raised to ≥0.7 IU/mL in at least one TB antigen minus the nil tube value. Nine (60%) of the 15 positive QFT-PD that were negative using QFT-PT remained QFT-PD positive when the cutoff was raised to a conservative level of ≥0.7 IU/mL. There was a significant difference between QFT-PD and QFT-PT for the absolute difference from 0.35 IU/mL for TB2 background corrected values (median 0.31 [IQR 0.17, 0.50] versus median 0.18 [IQR 0.10, 0.34], respectively, p = 0.03), but this difference was not seen in TB1 background corrected values (median 0.36 [IQR 0.22, 0.46] versus median 0.30 [IQR 0.21, 0.37], respectively, p = 0.18; Fig 2).

Using the cut-off of a positive assay ≥0.35 IU/mL, 60% of the positive QFT-PD results were positive due to both TB1 and TB2 compared to the 53% of positive QFT-PT (Table 3). When the cut-off is raised to ≥0.70 IU/mL, little difference in the proportion of assay results positive due to TB1 and TB2 for QFT-PD and QFT-PT (52% versus 54%). Raising the cutoff for positivity to ≥0.70 IU/mL causes a large reduction in the number of positive QFT-PT assays (n = 51 versus n = 26) indicating that almost half of the positive QFT-PT were near the cutoff (Table 3).

**Table 3:** QFT-P assay positivity based on antigen tube.

	Positive if ≥0.35 IU/mL				Positive if ≥0.70 IU/mL			
	Positive Results	TB1 only	TB2 only	TB1 and TB2	Positive Results	TB1 only	TB2 only	TB1 and TB2
	*n*	*n* (%)	*n* (%)	*n* (%)	*n*	*n* (%)	*n* (%)	*n* (%)
QFT-PD	35	7 (20.0)	7 (20.0)	21 (60.0)	25	7 (28.0)	5 (20.0)	13 (52.0)
QFT-PT	51	5 (9.8)	19 (37.3)	27 (52.9)	26	4 (15.4)	8 (30.8)	14 (53.8)

Positivity based on background (nil IFN-γ measure) corrected values.

### Risk factors

For HCWs with no additional TB risk factors (*n* = 115), there was 93.9% agreement (108/115) between the QFT-G and QFT-PD assays, 93.0% (107/115) agreement between the QFT-G and QFT-PT assays, and 87.0% (100/115) between the QFT-PD and QFT-PT assays (Table 4). For HCWs with one or more TB risk factors, there was 90.2% agreement (148/164) between the QFT-G and QFT-PD assays, 89.6% agreement (147/164) between the QFT-G and QFT-PT assays, and 84.8% agreement (139/164) between the QFT-PD and QFT-PT assays (Table 4).

**Table 4:** Percent agreement of QFT-G, QFT-PD, and QFT-PT by the number of risk factors.

	Number of Risk Factors^a^		
	0	1	≥2
Total (n = 279)	115	107	57
	*n* (%)	*n* (%)	*n* (%)
QFT Gold versus QFT + Direct	108 (93.9)	96 (89.7)	52 (91.2)
QFT Gold versus QFT + Transfer	107 (93.0)	94 (87.9)	53 (93.0)
QFT+ Direct versus QFT + Transfer	100 (87.0)	91 (85.0)	48 (84.2)

^a^ Analyzed risk factors included: having a prior positive TST or IGRA, previous treatment for TBI, history of autoimmune disease, taking immunosuppressive drugs, or receiving a vaccine within 6 weeks prior to having the QFT. One participant was removed from this analysis for not giving enough blood to complete the QFT-P assays

The analysis of risk factors for having a positive QFT assay found that daily consumption of green tea was significantly associated with having a positive QFT-G (OR: 4.84, *P* = 0.03), and receiving a vaccine within 6 weeks prior to the assay was significantly associated with a positive QFT-PD (OR = 6.09, *P* = 0.004) and QFT-PT (OR = 6.02, *P* = 0.004; Table 5).

**Table 5:** Risk factors for a positive assay result.

	QFT-G			QFT+D			QFT+T		
Covariate	Unadjusted Odds Ratio^a^	Adjusted Odds Ratio^a^	Adjusted p-value	Unadjusted Odds Ratio^a^	Adjusted Odds Ratio^a^	Adjusted p-value	Unadjusted Odds Ratio^a^	Adjusted Odds Ratio^a^	Adjusted p-value
Age									
<30	Ref.^b^			Ref.			Ref.		
31–64	0.99 (0.38–2.59)			1.28 (0.50–3.28)			1.37 (0.60–3.14)		
65+	3.11 (0.51–18.99)			3.11 (0.51–18.98)			2.7 (0.45–16.34)		
Gender									
Female	Ref.			Ref.			Ref.		
Male	0.83 (0.37–1.87)			0.89 (0.41–1.93)			0.87 (0.45–1.69)		
Pregnant									
Yes	-			1.23 (0.14–10.61)			0.82 (0.10–7.06)		
No	Ref.			Ref.			Ref.		
Ethnicity									
White	Ref.			Ref.			Ref.		
AA/Black	0.42 (0.08–2.09)			0.32 (0.07–1.56)			0.84 (0.29–2.44)		
Asian	1.23 (0.47–3.20)			1.00 (0.42–2.42)			1.39 (0.64–3.03)		
Other	1.82 (0.49–6.84)			2.28 (0.72–7.22)			1.09 (0.31–3.79)		
Hispanic									
Yes	2.01 (0.80–5.08)			1.31 (0.51–3.42)			1.23 (0.53–2.88)		
No	Ref.			Ref.			Ref.		
US-Born									
Yes	Ref.			Ref.			Ref.		
No	0.9 (0.35–2.36)			1.15 (0.45–2.96)			1.14 (0.49–2.62)		
Received BCG Vaccine									
Yes	1.59 (0.57–4.42)			1.46 (0.57–3.78)			1.06 (0.50–2.26)		
No	Ref.			Ref.			Ref.		
Don't know/Prefer not to answer	1.07 (0.19–5.87)			1.91 (0.49–7.40)			0.96 (0.28–3.33)		
Result Last TST									
Positive	2.23 (0.78–6.37)	0.52 (.11–2.57)	0.425	3.14 (1.13–8.69)	2.23 (0.54–9.31)	0.270	2.6 (1.12–6.04)	1.99 (0.57–6.95)	0.280
Negative	Ref.	Ref.		Ref.	Ref.		Ref.	Ref.	
Result Last TB Blood Test									
Positive	20.11 (4.92–82.25)	7.82 (0.74–82.92)	0.088	8.33 (2.10–33.04)	1.78 (0.22–14.70)	0.591	20.75 (4.95–86.99)	2.96 (0.36–24.26)	0.312
Negative	Ref.	Ref.		Ref.	Ref.		Ref.	Ref.	
Ever taken treatment for TBI									
Yes	0.73 (0.16–3.27)	1.68 (0.16–17.43)	0.666	0.58 (0.13–2.59)	0.98 (0.18–5.26)	0.983	0.41 (0.09–1.79)	0.34 (0.04–3.04)	0.336
No	Ref.	Ref.		Ref.	Ref.		Ref.	Ref.	
Medical Conditions									
Diabetes	0.81 (0.18–3.65)			2.25 (0.77–6.56)			1.52 (0.53–4.34)		
Autoimmune	0.68 (0.09–5.41)			0.58 (0.07–4.57)			-		
Medications									
Corticosteroids	-			1.04 (0.12–8.70)			-		
Smoke									
Yes	3.76 (0.69–30.33)			-			0.85 (0.10–7.22)		
No	Ref.			-			Ref.		
Vaccinated 6 weeks prior to assay									
Yes	5.40 (2.28–12.77)	3.43 (0.64–18.35)	0.150	3.35 (1.52–8.30)	6.09 (1.76–21.02)	0.004	4.77 (2.23–10.24)	6.02 (1.76–20.60)	0.004
No	Ref.	Ref.		Ref.	Ref.		Ref.	Ref.	
Viral illness within 6 weeks									
Yes	0.88 (0.11–7.15)			1.67 (0.35–8.09)			0.56 (0.7–4.50)		
No	Ref.			Ref.			Ref.		
Regularly consume green tea									
Yes	3.78 (1.70–8.40)	4.84 (1.16–20.17)	0.030	2.29 (1.08–4.84)	2.13 (0.67–6.80)	0.200	1.92 (0.98–3.77)	2.03 (0.66–6.22)	0.218
No	Ref.	Ref.		Ref.	Ref.		Ref.	Ref.	

^a^Odds Ratio (95% Confidence Interval)

^b^Ref. = Reference category

## Discussion

Within a select population of higher than average risk, HCWs in a low TB incidence country, the overall agreement between the QFT-G and the QFT-P, regardless of blood collection method was high, but there was lower agreement found between the QFT-PD and the QFT-PT. More positive results were found using the QFT-P than the QFT-G, and more positive results were found with the QFT-PT than the QFT-G and QFT-PD. Overall the agreement between the QFT-G, QFT-PD and QFT-PT is 85% or greater. This is in high agreement; however, when comparing the 3 different assays’ percent agreements, the QFT-PD and QFT-PT agreement were 6% lower than either the QFT-G or to either QFT-P assays. This difference is most likely due to the inherent variability of an immune-based assay being used in real world conditions. In addition, the sample size in this study was 299 and a larger sample size might show a greater agreement overall in the percent agreement between the QFT-PD and QFT-PT. Metcalfe et al. reported the within subject variability of the QFT-G for individuals in a low TB incidence setting was ± 0.6 IU/mL [16]. QFT results near the cutoff zone (0.35 IU/mL) can convert or revert test results upon re-testing. This may account for some of the discordant test results between the assays. When the cutoff for a positive QFT-P was raised to 0.7 IU/mL, the percent agreement between the QFT-PD and QFT-PT rose (85% versus 94%), and the agreement between the QFT-PT, QFT-PD, and the QFT-G increased when using the conservative definition of TB1 and TB2 results both ≥0.35 IU/mL when compared to at least one antigen tube with a value ≥0.35 IU/mL [17].

A large scale study comparing QFT-G and QFT-PD among U.S. HCWs reported an overall agreement between the two tests at 96% [8], which is similar to the percent agreement found in this study (91%). QFT-G, QFT-PD, and QFT-PT assays showed high percent agreement, but low κ reflecting poor agreement (0.56, 0.59, and 0.37) in the current study. The positivity among the large cohort of HCWs was found to be 4% with QFT-G and 6% with QFT-PD [8]. The positive QFT-G results and negative QFT-PD results were seen in 1% of HCWs, and negative QFT-G results and positive QFT-PD results were seen in 3% of HCWs [8]. These findings were similar to those seen in the current study (3% and 5%).

According to Feinstein et al, κ may be affected by the prevalence of test results (17). The low κ may be partially accounted for by the low prevalence of positive and indeterminate test results. κ underestimates agreement on rare outcomes. When percent agreement by chance (p_e_) is high, the calculated κ values indicate low agreement [18].

Discrepant results between the QFT-G and the QFT-P have been seen in other studies. Hoffmann et al. reported nine out of 163 patients tested with both assays had discordant results, and three (33%) of the cases had positive QFT-G results and negative QFT-P test results, while the other six cases were QFT-G negatives with positive QFT-P results [4]. A study among migrant students in Germany found that QFT-PT had a conversion rate of 4% and a reversion rate of 7% [19]. A large multi-center study conducted in Netherlands and Belgium found 50 of 1031 (5%) discordant test results between QFT-G and QFT-P assays, and 60% of these discordant results were in the borderline range of 0.25–0.8 IU/mL [6].

Several pre-analytical factors have been associated with indeterminate QFT-G results. These factors can affect the amount of IFN-γ measured in the assay and include duration between blood draw and incubation [15, 20, 21], blood volume [1, 15], tube shaking [1], and ELISA batching [15]. Vigorous shaking has been found to increase the median IFN-γ measured in the nil and TB Ag tubes [1]. Other factors that have been shown to affect the QFT-G include environmental factors such as pre-incubation temperature [22, 23] and season [24–26]. The greater frequency of positive assay results in the QFT-PT assay compared to the QFT-PD may have been caused by the increased amount of agitation sustained by the blood in the QFT-PT assay due to the transfer to the assay tubes compared to the QFT-PD blood. The amount of positive results seen with the QFT-PT assay was reduced when the cutoff was raised to 0.7 IU/mL potentially reducing the number of false positive results.

Another source of variability between the QFT-G and QFT-P results may be the standard curve used. The QFT-G assays were analyzed with an eight-point standard curve, but the two QFT-P assays were analyzed using a four-point standard curve per the instructions in the FDA-approved package insert. Nemes et al. reported finding that QFT-G samples analyzed with an eight-point standard curve had significantly higher IFN-γ values than samples analyzed with four-point standard curves [15]. The QFT-G and QFT-PD used the same positive and negative control in this study. The IFN-γ was estimated using the 8 point and 4 point standard curves for the QFT-G and QFT-PD assays, respectively. The standard curve used was not believed to have had an effect on the quantitative results of the assays.

Bittel et al. analyzed the differences in quantitative and qualitative QFT-G results between direct in-tube phlebotomy and blood collection using a LiHp tube [27]. Of the 107 HCWs screened, 98% had concordant qualitative results between the direct and transferred QFT-G [27]. A statistically significant difference was found between nil and mitogen IFN-γ measurements with the different phlebotomy methods indicating that the transfer affected the amount of IFN-γ being produced by PBMCs in the transferred tubes.

Different factors were identified as being associated with a positive QFT-G and QFT-P. Participants that claimed to regularly consume green tea reported drinking a median one cup per day (IQR: 1–2 cups). Green tea consumption was added to the analysis as a potential risk factor due to anecdotal evidence that some of our subjects who consumed green tea regularly had discrepant IGRA results. Compounds in green tea and thus green tea consumption have been found to affect IFN-γ production [28]. Because our HCW population has a high proportion of individuals of Asian descent we chose to include a green tea consumption question into our TB risk questionnaire to see if we were able to identify an association between green tea consumption and positive QFT results. There is certain evidence that green tea can increase the amount of IFN-γ secreted by splenocytes from mice treated with green tea extract [28], but there has been no systematic research on the effects of vaccines prior to administering a TST or an IGRA. Of the 37 participants that received a vaccine within 6 week prior to having phlebotomy for the QFT assays, 20 (54%) received a flu vaccine. There is evidence that influenza vaccination reduces the risk of TB incidence among the elderly [29]. It has been shown that there is an increase in IFN-γ producing NK cells and CD8+ T cells after influenza vaccination, and PBMCs cultured with influenza vaccine produced a greater amount of IFN-γ post-exposure compared to pre-exposure to the vaccine [30]. This evidence indicates that recent vaccination(s) can affect the amount of IFN-γ being produced. Excess IFN-γ production should ideally be controlled for in the assay by subtracting the baseline (unstimulated) IFN-γ measured, preventing false-positive results; however, the authors of this study are unaware of any studies conducted to determine how vaccines effect IGRAs directly. In addition, due to the small sample size of this project, these results should be interpreted cautiously.

This pilot study had several limitations. First, the low number of TB infected participants and the low number of indeterminate results limits our ability to investigate the agreement between the two QFT tests. This also limits our ability to assess if phlebotomy method can lower the number of indeterminate results among patients undergoing TBI testing at our institution. Although certain concerns may be raised when the QFT-Plus tubes are drawn after the Mitogen tube was drawn, there are no restriction in the manufacturer’s packet insert regarding the specific order of blood tubes to be drawn during phlebotomy. Therefore, the potential bias caused by the order of blood tubes to be drawn, if any, would be minimal. The plasma for the QFT-G was run immediately while the plasma for the QFT-P assays were frozen per manufacturer’s protocol and run at a later date possibly introducing variability. The lack of a gold standard for TBI screening limits our ability to determine which assay is correct in the case of discrepant results.

In spite of limitations, the current study has many strengths. First, the study included data on risk factors for TBI and indeterminate IGRA results, and the availability of prior test results (TST and IGRA) for participants. Simultaneous testing of participants using the QFT-G, QFT-PD and QFT-PT minimized potential pre-analytical and analytical sources of variability. Last, the study took place during routine annual screening making the results more likely to be generalizable in other regularly screened groups in low incidence settings.

The QFT-P assay, no matter the FDA approved blood collection method, showed a high percent agreement with the QFT-G assay among a population of U.S. HCWs when compared to other studies reporting the agreement of the QFT-G and QFT-P; however, the Cohen’s κ coefficients of inter-rater agreement indicated that the agreement between the assays was only from fair to moderate. The QFT-PT assay had numerous potential false positive assay results compared to the QFT-PD assay, but a larger study is needed to determine how to control for this variability. The use of the conservative interpretation cutoff of 0.7 IU/ml for a positive test result accounted for over half of the discrepant results. Without a “gold-standard”, this study was unable to determine if the QFT-P was able to detect TBI with equal or greater sensitivity to the QFT-G. The option of collecting blood into a single LiHp tube prior to transferring the blood into assay tubes increases the ability of clinics and public health programs to customize their individual protocols to better meet their needs whether on-site or in the field; however, it is currently unknown how using this alternate blood collection method may affect the performance of the QFT-P assay.

## Supporting information

S1 File Data set. (XLS)

## References

[1] Gaur RL, Pai M, Banaei N. Impact of Blood Volume, Tube Shaking, and Incubation Time on Reproducibility of QuantiFERON-TB Gold In-Tube Assay. JClinMicrobiol. 2013;51(11):3521–6. 10.1128/jcm.01627-13 23966505 10.1128/JCM.01627-13 PMC3889728

[2] Barcellini L, Borroni E, Brown J, Brunetti E, Codecasa L, Cugnata F, et al First independent evaluation of QuantiFERON-TB Plus performance. European Respiratory Journal. 2016;47(5):1587–90. 10.1183/13993003.02033-2015 26869677 10.1183/13993003.02033-2015

[3] Yi L, Sasaki Y, Nagai H, Ishikawa S, Takamori M, Sakashita K, et al Evaluation of QuantiFERON-TB gold plus for detection of Mycobacterium tuberculosis infection in Japan. Scientific Reports. 2016;6 10.1038/srep30617 27470684 10.1038/srep30617 PMC4965764

[4] Hoffmann H, Avsar K, Göres R, Mavi S-C, Hofmann-Thiel S. Equal sensitivity of the new generation QuantiFERON-TB Gold plus in direct comparison with the previous test version QuantiFERON-TB Gold IT. Clinical Microbiology and Infection. 2016;22(8):701–3. 10.1016/j.cmi.2016.05.006 27184875 10.1016/j.cmi.2016.05.006

[5] Petruccioli E, Vanini V, Chiacchio T, Cuzzi G, Cirillo D, Palmieri F, et al Analytical evaluation of QuantiFERON-Plus and QuantiFERON-Gold In-tube assays in subjects with or without tuberculosis. Tuberculosis. 2017;106:38–43. 10.1016/j.tube.2017.06.002 28802403 10.1016/j.tube.2017.06.002

[6] Pieterman E, Lung FL, Verbon A, Bax H, Ang C, Berkhout J, et al A multicentre verification study of the QuantiFERON®-TB Gold Plus assay. Tuberculosis. 2018;108:136–42. 10.1016/j.tube.2017.11.014 29523314 10.1016/j.tube.2017.11.014

[7] Takasaki J, Manabe T, Morino E, Muto Y, Hashimoto M, Iikura M, et al Sensitivity and specificity of QuantiFERON-TB Gold Plus compared with QuantiFERON-TB Gold In-Tube and T-SPOT. TB on active tuberculosis in Japan. Journal of Infection and Chemotherapy. 2017;24(3):188–92. 10.1016/j.jiac.2017.10.009 29108749 10.1016/j.jiac.2017.10.009

[8] Moon H-W, Gaur RL, Tien SS-H, Spangler M, Pai M, Banaei N. Evaluation of QuantiFERON®-TB Gold-Plus in Healthcare Workers in a Low-Incidence Setting. JClinMicrobiol. 2017;55(6):1650–7. 10.1128/JCM.02498-16 PMC5442521 28298455

[9] Mazurek GH, LoBue PA, Daley CL, Bernardo J, Lardizabal AA, Bishai WR, et al Comparison of a whole-blood interferon γ assay with tuberculin skin testing for detecting latent Mycobacterium tuberculosis infection. JAMA. 2001;286(14):1740–7. 11594899 10.1001/jama.286.14.1740

[10] Menzies D, Pai M, Comstock G. Meta-analysis: new tests for the diagnosis of latent tuberculosis infection: areas of uncertainty and recommendations for research. Annals of Internal Medicine. 2007;146(5):340–54. 17339619 10.7326/0003-4819-146-5-200703060-00006

[11] Zwerling A, van den Hof S, Scholten J, Cobelens F, Menzies D, Pai M. Interferon-gamma release assays for tuberculosis screening of healthcare workers: a systematic review. Thorax. 2012;67(1):62 10.1136/thx.2010.143180 . 21228420 10.1136/thx.2010.143180

[12] QuantiFERON®-TB Gold Plus (QFT®-Plus) ELISA Package Insert. Hilden, Germany: 2016 02/2016. Report No.

[13] QuantiFERON®-TB Gold Plus (QFT®-Plus) Blood Collection Tubes Package Insert. Germantown Rd, Germantown MD, USA: 2017 08/2017. Report No.

[14] Connelly LM. Pilot studies. Medsurg Nursing. 2008;17(6):411–3. 19248407

[15] Nemes E, Rozot V, Geldenhuys H, Bilek N, Mabwe S, Abrahams D, et al Optimization and Interpretation of Serial QuantiFERON Testing to Measure Acquisition of Mycobacterium tuberculosis Infection. American Journal of Respiratory and Critical Care Medicine. 2017;196(5):638–48. 10.1164/rccm.201704-0817OC 28737960 10.1164/rccm.201704-0817OC PMC5620669

[16] Metcalfe JZ, Cattamanchi A, McCulloch CE, Lew JD, Ha NP, Graviss EA. Test variability of the QuantiFERON-TB gold in-tube assay in clinical practice. American journal of respiratory and critical care medicine. 2013;187(2):206–11. 10.1164/rccm.201203-0430OC 23103734 10.1164/rccm.201203-0430OC PMC3570654

[17] Agarwal S, Nguyen D, Lew JD, Graviss EA. Phlebotomy Methods May Affect QuantiFERON Gold Plus Assay Results. AnnAmThoracSoc. Forthcoming.

[18] Feinstein AR, Cicchetti DV. High agreement but low kappa: I. The problems of two paradoxes. Journal of clinical epidemiology. 1990;43(6):543–9. 2348207 10.1016/0895-4356(90)90158-l

[19] Knierer J, Morales EG, Schablon A, Nienhaus A, Kersten J. QFT-Plus: a plus in variability?–Evaluation of new generation IGRA in serial testing of students with a migration background in Germany. Journal of Occupational Medicine and Toxicology. 2017;12(1):1. 28070206 10.1186/s12995-016-0148-z PMC5216544

[20] Herrera V, Yeh E, Murphy K, Parsonnet J, Banaei N. Immediate incubation reduces indeterminate results for QuantiFERON-TB Gold in-tube assay. JClinMicrobiol. 2010;48(8):2672–6. 10.1128/JCM.00482-10 PMC2916573 20519472

[21] Doberne D, Gaur RL, Banaei N. Preanalytical delay reduces sensitivity of QuantiFERON-TB gold in-tube assay for detection of latent tuberculosis infection. JClinMicrobiol. 2011;49(8):3061 10.1128/JCM.01136-11 . 21697332 10.1128/JCM.01136-11 PMC3147723

[22] Jarvis J, Gao Y, de Graaf H, Hughes S, Allan RN, Williams A, et al Environmental temperature impacts on the performance of QuantiFERON-TB Gold In-Tube assays. Journal of Infection. 2015;71(2):276–80. 10.1016/j.jinf.2015.04.004 25869537 10.1016/j.jinf.2015.04.004

[23] Shanaube K, De Haas P, Schaap A, Moyo M, Kosloff B, Devendra A, et al Intra-assay reliability and robustness of QuantiFERON®-TB Gold In-Tube test in Zambia. The international journal of tuberculosis and lung disease. 2010;14(7):828–33. 20550764

[24] Griffin ML, Agarwal S, Murphy MZ, Teeter LD, Graviss EA. Influence of Seasonality and Circulating Cytokines on Serial QuantiFERON Discordances. Tuberculosis research and treatment. 2018;2018. 10.1155/2018/6731207 PMC5867601 29721337

[25] Agarwal S, Nguyen DT, Graviss EA. Season Is Associated with Interferon Gamma Measured in QuantiFERON Gold In-Tube Test. Open forum infectious diseases. 2017;4(Suppl 1):S621.

[26] Tebruegge M, Curtis N, Clifford V, Fernandez-Turienzo C, Klein N, Fidler K, et al Seasonal variation in the performance of QuantiFERON-TB Gold In-Tube assays used for the diagnosis of tuberculosis infection. Tuberculosis. 2018;110:26–9. 10.1016/j.tube.2018.03.002 29779769 10.1016/j.tube.2018.03.002

[27] Bittel P. Sample Collection Procedures and Impact on QuantiFERON®-Gold in Tube Results. Journal of Diagnostic Techniques and Biomedical Analysis. 2015;5(1):2.

[28] Kuo C-L, Chen T-S, Liou S-Y, Hsieh C-C. Immunomodulatory effects of EGCG fraction of green tea extract in innate and adaptive immunity via T regulatory cells in murine model. Immunopharmacology and immunotoxicology. 2014;36(5):364–70. 10.3109/08923973.2014.953637 25151997 10.3109/08923973.2014.953637

[29] Yen Y-F, Pan S-W, Su VY-F, Chuang P-H, Feng J-Y, Su W-J. Influenza Vaccination and Incident Tuberculosis among Elderly Persons, Taiwan. Emerging infectious diseases. 2018;24(3):498 10.3201/eid2403.152071 29460733 10.3201/eid2403.152071 PMC5823323

[30] Long BR, Michaelsson J, Loo CP, Ballan WM, Vu B-AN, Hecht FM, et al Elevated frequency of gamma interferon-producing NK cells in healthy adults vaccinated against influenza virus. Clinical and Vaccine Immunology. 2008;15(1):120–30. 10.1128/CVI.00357-07 18003818 10.1128/CVI.00357-07 PMC2223854

